# Reply to: Antibiotics and hexagonal order in the bacterial outer membrane

**DOI:** 10.1038/s41467-023-40276-z

**Published:** 2023-08-09

**Authors:** Selen Manioglu, Seyed Majed Modaresi, Johannes Thoma, Sarah A. Overall, Gregory Upert, Anatol Luther, Alexander B. Barnes, Daniel Obrecht, Daniel J. Müller, Sebastian Hiller

**Affiliations:** 1 Department of Biosystems Science and Engineering, Eidgenössische Technische Hochschule (ETH) Zürich, Mattenstrasse 26, 4058 Basel, Switzerland; 2 Biozentrum, University of Basel, Spitalstrasse 41, 4056 Basel, Switzerland; 3 Department of Chemistry and Molecular Biology, University of Gothenburg, Göteborg, 405 30 Göteborg, Sweden; 4 Laboratory of Physical Chemistry, ETH Zurich, 8093 Zurich, Switzerland; 5 Spexis AG, 4123 Allschwil, Switzerland; 6 Bachem AG, 4416 Bubendorf, Switzerland

**Keywords:** Bacteria, Atomic force microscopy

**replying to** G. Benn et al. *Nature Communications* 10.1038/s41467-023-40275-0 (2023)

Benn et al. comment that the hexagonal lattices observed in outer membrane (OM) patches upon the addition of polymyxin might be formed by outer membrane proteins (OMPs). They assume that lipopolysaccharides (LPS) protrude above the OMPs, which might hide the OMPs and prevent them from being imaged by AFM. Furthermore, they speculate that polymyxin somehow orders the LPS molecules, such that the hidden OMP lattice is revealed upon polymyxin addition. Indeed this, or that polymyxin induces other biophysical changes to the membrane, are possible scenarios that could follow from our study^1^ and that will require further investigation, possibly with higher temporal or spatial imaging modalities.

It has been established for decades that OMPs can assemble different densely packed assemblies in lipid membranes, the geometry of which depends on multiple parameters including lipid composition, electrolytes, and incubation protocol^2^. Our membrane patches of outer membrane vesicles (OMVs) natively released from *Escherichia coli* and imaged by high-resolution AFM show regions of different OMP densities, that is, with dense assembly of OMPs, sparse assembly of OMPs, or almost devoid of any OMPs (Fig. 1a–f). From such OMV membranes, as well as from OmpF trimers artificially reconstituted into phospholipid membranes as crystalline lattices, one can measure that OMPs protrude with their extracellular domains considerably from the membrane surface so that they can be directly imaged by the scanning AFM tip (Fig. 1). In our OMVs, the densely packed regions of OMPs are rarer compared to regions devoid of OMPs, which presumably are only lipids because they protrude less than OMPs.

**Fig. 1:**
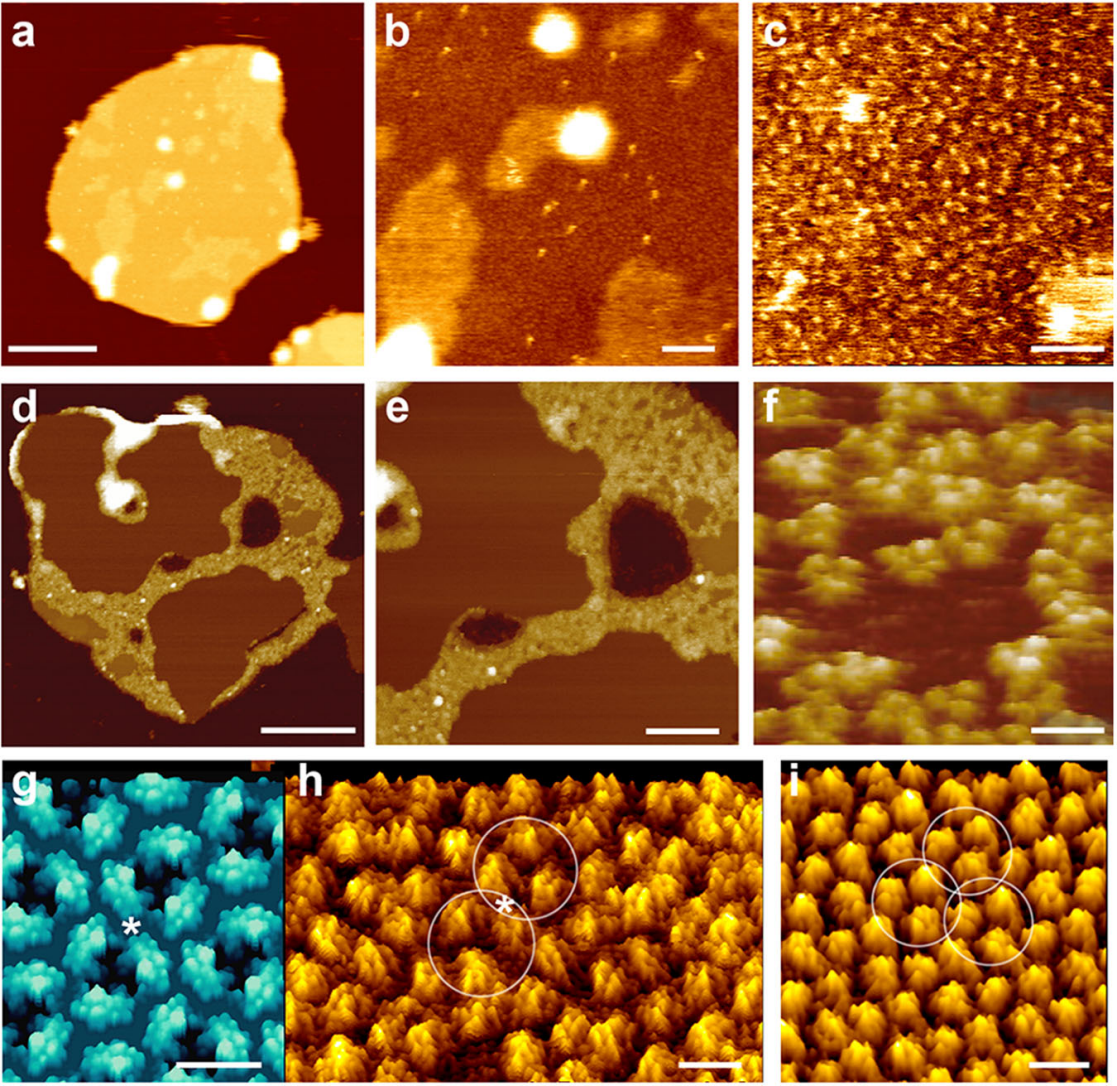
Outer membrane protein assemblies imaged by high-resolution atomic force microscopy (AFM). **a**–**c** AFM height images of OMVs collected from *E. coli* overexpressing OMPs. **a** Overview AFM height image of an OMV enriched in the OMP Tsx^8^. Upon adsorption to mica, the OMVs opened as single layered membrane patches. **b** Region from (**a**) imaged at higher resolution. The membrane contains densely distributed particles protruding from the membrane. **c** High-resolution height image revealing OMPs (single protrusions) in densely packed arrangements. **d**, **e** Overview AFM height image of an OMV enriched in the OMP BamA^9^. The densely packed areas of BamA appear higher (yellow, heights of 10–15 nm) than the surrounding membrane (heights of 5–8 nm). **f** Extracellular surface of OmpF trimers in a membrane containing lipopolysaccharide (LPS) and *E. coli* lipids^7^. **g** Extracellular surface of the atomic model of OmpF trimers rendered at 3 Å (**h**, **i**) High-resolution AFM height images of 2D porin OmpF lattices assembled in lipid membranes containing LPS and phospholipids^5^. The extracellular domains formed by the long OmpF loops protrude by 1.3 nm from the membrane. Shown are OmpF trimers assembled in rectangular (**h**, 13.5 nm × 8.2 nm) or trigonal (**i**, 8.2 nm) packing arrangements. AFM images represent height images taken in buffer solution. The brightness range of the AFM height images corresponds to a vertical range of 16 nm (**a**), 4 nm (**b**), 1 nm (**c**), 30 nm (**d**, **e**), ≈ 1-2 nm (**f**) and 1.5 nm (**h**, **i**). Height images (**f**, **h**, **i**) are displayed as perspective views. Scale bars, 200 nm (**a**), 50 nm (**b**) and 20 nm (**c**), 160 nm (**d**), 130 nm (**e**), 10 nm (**f**) and 5 nm (**g**–**i**). Images were taken from (**a**–**c**) ref. ^8^, (**d**, **e**) ref. ^9^, (**f**) ref. ^7^, and (**g**–**i**) ref. ^5^.

Upon addition of polymyxins to such OMV patches, we observed that most of the large surface areas that were empty before become covered with hexagonal lattices.

This formation occurs in a highly similar manner, independently of the OMP composition of the membrane patches. OMVs collected from *E. coli* wild-type strain MG1655 (Fig. 2), as well as OMVs from *E. coli* strain BL21(DE3)omp8, from which the most abundant OMPs including OmpF, OmpC and others have been genetically deleted, show the same lattice structures. The OMV membranes of strain BL21(DE3)omp8 comprise primarily lipids, as indicated by low- and high-resolution AFM height images (Fig. 1d, e), and in line with the high capacity of this strain to incorporate overexpressed OMPs^3^. We also observe the same structures when OMVs collected from strain BL21(DE3)omp8 are specifically enriched with monomeric OmpG or BamA. Therefore, the formation of hexagonal lattices does not seem to require a specific OMP to be present in high abundance. Nevertheless, it is also clear that the OMPs initially present in the membrane are somehow included into the hexagonal lattices because we frequently observe entire membrane patches covered by lattices upon polymyxin addition (Fig. 2).

**Fig. 2:**
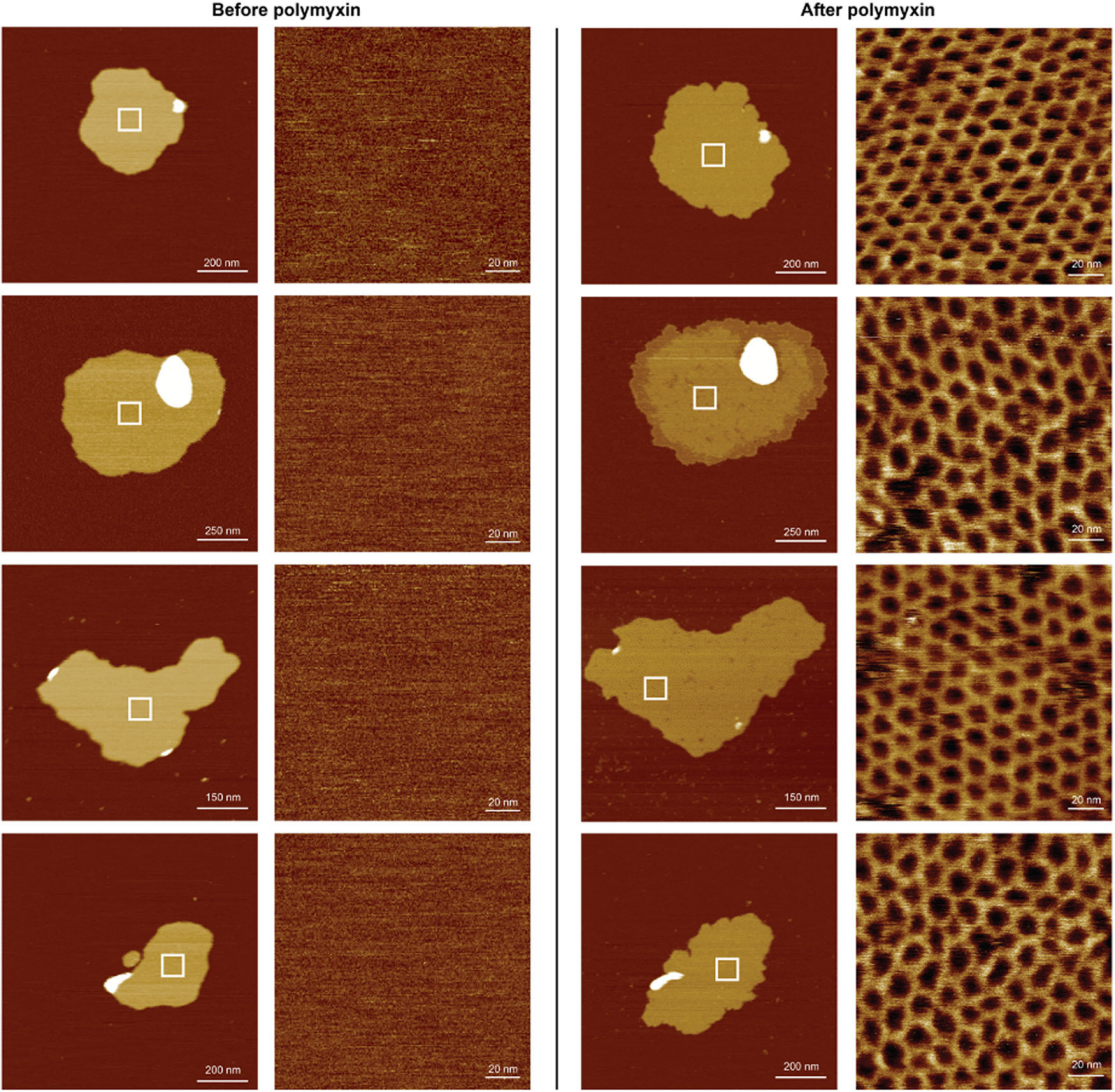
The addition of polymyxin to OMV membranes induces hexagonal assemblies. AFM height images of multiple membranes of OMVs recorded in buffer solution at room temperature. Each OMV membrane is shown before and after the addition of polymyxin. White boxes indicate where AFM height images were recorded to show the appearance of the membrane surface at higher resolution. The absence of structural details indicates that the membrane regions were either devoid of OMPs and contained mainly lipids or that OMPs could not be resolved. Indicative of adding polymyxin to OMV membranes is that they reduce thickness, increase surface area and are covered entirely with hexagonal lattices. OMVs from *E. coli* MG1655 WT strain, the AFM sample preparation, and the AFM imaging were prepared as described^1^. The brightness range of the AFM height images corresponds to a vertical range of 18 nm (overviews) and 2 nm (zoom ins). Scale bars, 200 nm (overviews) and 20 nm (zoom ins).

Interestingly, in strains MG1655 ∆waaG and MG1655 ∆waaC, which have a modified LPS but wild-type protein composition, we observe alterations in the polymyxin-induced crystalline structures compared to wild-type MG1655. If the lattice is indeed involving OMPs, their precise arrangement is mediated by the LPS molecules and wild-type LPS is required for the well-ordered hexagonal lattices. Also, in these strains the polysaccharides are genetically shortened, but no lattice is observed in the absence of polymyxin.

Regarding the alternative model proposed by Benn et al, several points will need to be clarified: Firstly, how is stealthing of OMP lattices by LPS possible? LPS would need to protrude higher than the OMPs, but already in the absence of polymyxin we and others observe OMPs to protrude higher than the imaged membrane surface devoid of OMPs.

In addition, what is the identity of the OMP forming the hidden lattices? It seems unlikely that these are OmpF lattices. OmpF trimers can assemble different lattices, of which the hexagonal one shows a 8–9 nm periodicity^2,4^. Imaging trimeric OmpF reconstituted in lipid membranes or OMVs at sufficiently well-adjusted AFM imaging conditions can provide a resolution that allows for unambiguous fitting of the X-ray structure (Fig. 1f–i). The trimers consistently show large extracellular domains that protrude by ≈0.8–1.3 nm from the membrane surface and appear at distances of ≈4 nm^4–7^. Therefore, while some of the lattice parameters match, some other parameters do not match at all. Also, the lattices were observed in the *E. coli* strain BL21(DE3)omp8, which is devoid of OmpF.

Furthermore, how is the lattice hidden in the case of strains with strongly truncated LPS? Strains MG1655 ∆waaG and MG1655 ∆waaC have polysaccharide chains that are so short that it is hard to imagine how they can cover entire OMPs.

And finally, how does the addition of polymyxin order the LPS to reveal the lattices?

Overall, it seems that there is still a lot to learn about the ultrastructure of bacterial outer membranes and their interactions with lipid-targeting antibiotics.

## Methods

### AFM imaging and analysis

OMVs produced from different E. coli MG1655 strains were adsorbed onto freshly cleaved mica for 15 min in DPBS buffer at room temperature. After adsorption, the sample was gently washed with fresh DPBS buffer for five times to remove non-adsorbed OMVs. Then OMVs were imaged using force-distance curve-based AFM (FD-based AFM) performed with an AFM (Nanoscope Multimode 8, Bruker) operated in PeakForce Tapping mode in buffer solution (DPBS) at room temperature. The AFM was equipped with a 120 μm piezoelectric scanner and fluid cell. The images were recorded using two different AFM cantilevers: PEAKFORCE‐HiRs‐F‐A (Bruker) with a nominal spring constant of 0.4 N/m, a resonance frequency of ≈165 kHz in liquid, and a sharpened silicon tip with a nominal radius of ≈1 nm or SCANASYST-FLUID + (Bruker) with a nominal spring constant of 0.7 N/m, a resonance frequency of ≈150 kHz in liquid, and a sharpened silicon tip with a nominal radius of ≈2 nm. Before imaging, cantilevers were calibrated by ramping on the mica surface and the thermal tuning method. Images were recorded at 2 kHz oscillation frequency, by applying an imaging force of 100–120 pN with a vertical amplitude of 30 nm. The AFM was placed inside a home‐built acoustic isolated and temperature‐controlled box. Images were collected on a timescale of minutes. Raw AFM images were processed using the AFM analysis software Nanoscope v.1.8 for levelling and flattening.

## Data Availability

This response does not contain original data. The data shown has been previously published and is referenced accordingly.
